# The basal ganglia, the ideal machinery for the cost-benefit analysis of action plans

**DOI:** 10.3389/fncir.2013.00121

**Published:** 2013-07-22

**Authors:** Eun Jung Hwang

**Affiliations:** Division of Biology and Biological Engineering, California Institute of TechnologyPasadena, CA, USA

**Keywords:** action-selection, decision-making, reward, effort, optimal control

## Abstract

Basal ganglia dysfunction causes profound movement disorders, often attributed to imbalance between direct and indirect pathway activity in the sensorimotor basal ganglia. In the classical view, the direct pathway facilitates movements, whereas the indirect pathway inhibits movements. However, the recent finding of co-activation of the two pathways during movement challenges this view. Reconciling the new finding with the body of evidence supporting the classical view, this perspective proposes that the direct pathway computes the expected benefits of motor plans entering the basal ganglia, while the indirect pathway computes their expected costs. Thus, basal ganglia output combining the two pathway signals in a subtraction manner weighs benefits against costs, and endorses the plan with the best prospective outcome via feedback projections to the cortex. The cost-benefit model, while retaining the antagonistic roles of the two pathways for movements, requires co-activation of the two pathways during movement as both benefit and cost are computed for every movement. The cost-benefit model, though simple, accounts for a number of confounding results, and generates new focus for future research with testable predictions.

## Introduction

The basal ganglia (BG) are subcortical structures implicated in various neurological disorders including Parkinson’s disease, Huntington’s disease, obsessive-compulsive disorder, schizophrenia, and addictions (Steiner and Tseng, 2010). An understanding of BG circuits is essential for developing effective treatments for these disorders and for unraveling the neural basis of motor control, habit-formation, decision-making, and reinforcement learning. The BG contain two parallel circuits, so called direct and indirect pathways. Imbalanced activity of the two pathways has been linked to Parkinson’s disease and Huntington’s disease (Albin et al., 1989; Richfield et al., 1995; Mallet et al., 2006). However, the functional role of the two pathways is still under debate. The recent advent of cell-type specific intervention and recording techniques invigorated efforts to dissect the two pathways in greater detail. Unfortunately, such cutting-edge studies have so far reported confusing discoveries.

For instance, optogenetic activation of the indirect pathway caused bradykinesia (e.g., increased freezing and reduced locomotion), whereas activation of the direct pathway caused the opposite (Kravitz et al., 2010). This and similar causative studies support the prevailing classical model in the field that the direct pathway facilitates movements, whereas the indirect pathway inhibits movements (Durieux et al., 2009; Bateup et al., 2010). In contrast, optical recording of neuronal activity detected concurrent activation in the two pathways during normal movement, challenging the classical model which postulates less activity in the indirect pathway during movement than during rest (Cui et al., 2013). Perhaps even more confounding, repetitive optogenetic activation of the direct pathway following a voluntary movement reinforced that movement, whereas animals avoided movements that were followed by indirect pathway activation, emphasizing dissociative roles of the two pathways in reinforcement learning (Kravitz et al., 2012).

The growing influx of such conflicting and disconnected experimental results demands a new unifying model for the functional role of the two pathways, with the specificity that is necessary to make novel testable predictions and guide future research.

## Action selection

Of the many alternatives to the classical BG model (Mink, 1996; Gillies and Arbuthnott, 2000; Gurney et al., 2004), action selection models are the most prominent. It is well accepted that the anatomical architecture of the BG is fit for the function of selection. The BG receive massive inputs from various cortical and subcortical areas, process these inputs, and return the processed information back to where the inputs originated (Alexander et al., 1986; Alexander and Crutcher, 1990). Action selection models propose that the massive inputs contain competing action plans, the signal processing in the BG determines the re-entrant feedback signal for each action plan, and the action plan with facilitating feedback signal survives, while the others perish. In this view, the BG are a sophisticated action selection device rather than a gross movement generator or brake (Mink, 1996; Redgrave et al., 1999; Hikosaka et al., 2000; Frank, 2011).

**Figure 1:**
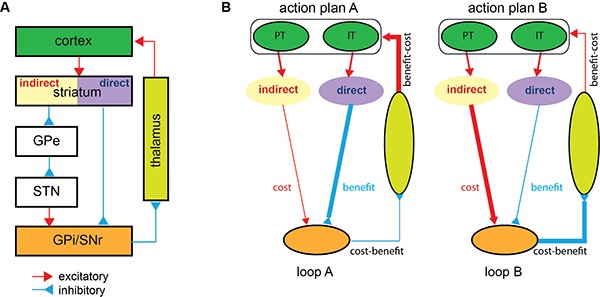
**(A)** The cortico-basal ganglia-cortico loop (Albin et al., 1989). Most cortical areas send excitatory projections to the striatum. The striatal projection neurons expressing D2 dopamine receptors transmit the cortical signal indirectly to the internal globus pallidus (GPi)/substantia nigra pars reticulate (SNr) via the external globus pallidus (GPe) and subthalamic nucleus (STN). The striatal projection neurons expressing D1 dopamine receptors transmit the cortical signal directly to the GPi/SNr. The GPi/SNr sends inhibitory projections to the thalamic nuclei, which then send excitatory projections back to the cortex. The red lines indicate excitatory connections, and the blue lines indicate inhibitory connections. Note that only principal pathways and not all identified connections are shown. **(B)** The cost-benefit model. An illustration of a simple scenario in which two action plans, A and B, compete. Two cortico-basal ganglia-cortico loops that are topologically organized, each linking a particular cortical ensemble with a particular set of basal ganglia neurons, operate in parallel. In loop A, (1) action plan A is represented by a set of intra-telencephalic (IT) and pyramidal tract (PT) neurons in the cortex, (2) indirect pathway neurons, preferentially processing PT neuronal input, produce the expected cost of plan A, and direct pathway neurons, preferentially processing IT neuronal input, produce the expected benefit of plan A, and (3) the basal ganglia output neurons combine the two pathways in a subtractive manner to represent the net cost-benefit of plan A. Likewise, in loop B, the basal ganglia output neurons represent the net cost-benefit of plan B. Action plan A producing the better prospective net value sustains through the strong re-entrant feedback signal from the basal ganglia to the cortex, whereas action plan B perishes. Although not depicted, the two pathways receive sensory cortical inputs so that the cost and benefit analysis draws on the sensory state information as well. GPe and STN are also omitted for brevity. The same color scheme as in **(A)** is used for different brain areas, and the line thickness indicates the signal strength.

Before introducing an elaborated model of action selection, it is necessary to review the principal synaptic connections in the BG. The main input structure of the BG, the striatum, receives glutamatergic, excitatory inputs from the cortex (Figure 1A). BG output nuclei send their GABAergic, inhibitory projections to the thalamic nuclei, which then send glutamatergic projections to primarily the same cortical areas from which the cortico-striatal inputs originated. Because BG output neurons have high spontaneous baseline activity, the thalamic target nuclei are normally inhibited. The excitatory cortical signals entering the BG propagate through direct and indirect pathways. The direct pathway consists of one GABAergic connection from the striatum to the output nuclei. The indirect pathway via two other nuclei consists of two GABAergic and one glutamatergic connections. Thus, direct pathway activation suppressing the activity of BG output neurons disinhibits the thalamic target, whereas indirect pathway activation intensifying output activity suppresses the target.

According to this synaptic map, action plans that activate the direct pathway would produce facilitating feedback signals and get selected for execution, while action plans activating the indirect pathway would produce suppressing feedback signals and get cancelled. The BG, therefore, can smartly arbitrate competing action plans by channeling a desirable action plan through the direct pathway but competing, unwanted plans through the indirect pathway. Such smart sorting may incorporate reinforcement learning mechanisms within the BG. For example, if the outcome of a particular action is better than expected (i.e., the reward prediction error is positive), cortico-striatal synapses transmitting that action plan to the direct pathway may be strengthened so that the same action will be more likely selected in the future under similar sensory and internal conditions. Indeed, dopaminergic neurons projecting to the striatum appear to encode reward prediction errors to some extent (Schultz et al., 1997), and dopamine dependent plasticity has been observed at cortico-striatal synapses (Shen et al., 2008). As such, action selection models were elegantly inferred from the bottom-up analysis of anatomy and physiology.

## Cost-benefit analysis

An outstanding question, though, is why a two-pathway structure is necessary given that the two pathways converge at the tonically firing output nuclei (Nambu, 2011) and thus, single pathway schemes seem sufficient to produce the re-entrant feedback in a prospective reward dependent manner. The answer may be sought using a top-down approach by specifying necessary computational elements to explain our action selection behaviors. Imagine you are going out for dinner tonight and have to choose a restaurant among many alternatives. Various aspects will be considered, including food tastes, dining atmospheres, prices, and distances. These are a list of benefits and costs associated with the available options. We and other animals avoid effortful actions if the benefit is held constant across action alternatives requiring different effort levels, yet we are willing to make effortful actions if higher benefits are predicted (Bautista et al., 2001; Stevens et al., 2005; Rudebeck et al., 2006; Gan et al., 2010). Thus, a critical process in action selection is to compute both benefits and costs for action alternatives and to weigh the benefits against costs. Various areas in the brain have been implicated for processing action costs or benefits, respectively, but it has not been clearly shown where and how the expected costs and benefits are measured against each other to affect action choice (Rudebeck et al., 2006; Croxson et al., 2009; Amemori and Graybiel, 2012).

A closer examination of the anatomy of the BG reveals that the two-pathway structure confers the necessary apparatus to perform such cost-benefit analysis for action selection. First, striatal neurons in the two pathways appear to receive different cortical inputs. Direct pathway striatal neurons preferentially receive cortical input from intra-telencephalic neurons that carry associative signals that are important for the computation of benefits, such as the context and abstract level action goals (Turner and DeLong, 2000; Lei et al., 2004). Indirect pathway striatal neurons receive greater inputs from the pyramidal-tract cortical neurons that carry the actual descending motor command signals (Lei et al., 2004). Motor command signals are most relevant for computing cost factors such as the energetic costs (efforts) and control risks of action plans (Todorov, 2000; Lei et al., 2004; Diedrichsen et al., 2010). Therefore, the direct pathway has access to information crucial for benefits, and the indirect pathway has access to information crucial for costs. Furthermore, as described earlier, activation of the two pathways exert opposite effects on the BG output (Figure 1A). In other words, the BG output takes the difference between the signals carried through the two pathways. This unique subtraction circuitry, combined with the distinct cortical input features, makes the two-pathway structure the ideal machinery for cost-benefit analysis.

Therefore this perspective hypothesizes that the sensorimotor BG direct pathway computes the expected benefits for action plans represented in the cortical input, while the indirect pathway computes their expected costs. The BG output combining the two pathways in a subtraction manner represents the net cost-benefit values. Then, due to the re-entrant feedback loops from the BG output to the cortex, the action with the highest prospective net value emerges as the winner (Redgrave et al., 1999). To better understand the model, imagine a situation where two action plans, A and B compete (Figure 1B). The cortical ensembles representing the two action plans form two cortico-BG-cortico loops, A and B, respectively. In loop A, BG direct and indirect pathways compute the expected benefit and cost for action A and BG output represents the prospective net value of action A. The re-entrant feedback facilitates or attenuates the cortical activity representing action A depending on its prospective value. Likewise, the cortical activity representing action B is modulated by its prospective value through loop B. Loop iterations, therefore, unequally modulate the two action representations in the cortex, leading to the selection of the action with the better prospective value.

In the computation of costs through the indirect pathway, various negative consequences of movements can be considered, including the energy expenditures (effort), control risks (e.g., motor errors, the loss of stability due to movements) (Harris and Wolpert, 1998), and punitive outcomes. Factors such as time and uncertainty that modulate the value of reward may or may not be processed through the indirect pathway. Temporal delays between movement and reward discount the value of reward. However, pharmacological activation of the indirect pathway failed to affect delay-dependent choice, whereas effort-dependent action choice was affected (van Gaalen et al., 2006; Salamone et al., 2007). In the case of uncertainty, it is unclear whether uncertainty, per se, has negative valence to be avoided (Platt and Huettel, 2008).

In the cost-benefit model, the BG continuously compute prospective values for instantaneous movement plans represented in the instantaneous cortical input. Theoretical and experimental studies suggest that motor commands are continuously evaluated and optimized moment-by-moment during movement using the latest sensory state information (Todorov, 2000; Shadmehr and Krakauer, 2008; Diedrichsen et al., 2010). The instantaneous action-value evaluation in the BG might underlie this moment-by-moment decision on motor commands. Compatible with this view, BG output activity shows task dependent modulation during movement (Mink, 1996). Furthermore, Huntington’s disease patients produce inappropriately exaggerated in-flight correction, indicating disrupted moment-by-moment decisions (Smith et al., 2000).

Despite its resemblance to old action selection models, the cost-benefit model bears nontrivial differences. Unlike the focused selection model (Mink, 1996) in which desirable actions channel through the direct pathway and undesirable actions through the indirect pathway, in the cost-benefit model every action plan enters both pathways. Moreover, in the cost-benefit model, the role of the indirect pathway is the expected-cost computation for potential motor plans, instead of a blanket inhibition of all motor plans as proposed in earlier action selection models (Gurney et al., 2004). Although the cost-benefit model may not come as a surprise to some in the field, the model for the first time articulates the idea of dissociative roles of the two pathways using computationally tractable parameters.

## The cost-benefit dependent action selection model

The cost-benefit model reconciles the seemingly conflicting findings between the causative and correlative studies described in the Introduction. In order to make optimal choices, both benefits and costs must be concurrently computed for potential motor plans. Even when only a single action plan is considered, that action should be better than “not moving at all” in terms of the net cost-benefit, to be executed. Therefore, both pathways, respectively computing the benefit and cost of the single plan, should be activated. The cost-benefit model can also explain the opposing effects of selective intervention between the two pathways. Indirect pathway activation should cause the rise of expected costs for all action plans, impeding the initiation and execution of movements. Likewise, direct pathway activation should cause the rise of expected benefits, justifying otherwise unexecuted costly movements. Similar accounts can be given to the opposing motor symptoms of Parkinson’s and Huntington’s diseases. Indirect pathway underactivity in Huntington’s disease (Reiner et al., 1988; Richfield et al., 1995) can be viewed as abnormal cost deflation for all action plans, resulting in an excess of spontaneous movements. Indirect pathway overactivity in Parkinson’s disease (Mallet et al., 2006) can be viewed as abnormal cost inflation, resulting in slowness, and lack of spontaneous movements. Supporting this view, Parkinson’s patients exhibit an abnormally severe sensitivity to the energy expenditure needed for movements although they are physically capable of making high cost movements (Mazzoni et al., 2007).

The cost-benefit model also provides a functional explanation for the dissociative learning effects between the two pathways (Hikida et al., 2010; Kravitz et al., 2012). Paired stimulations of cortical and striatal neurons can induce long-term potentiation at their glutamatergic synapses (Shen et al., 2008; Bateup et al., 2010). Therefore, repetitive direct pathway stimulation following a particular action over many trials could facilitate the cortical signal transmission representing that action in the direct pathway. The signal facilitation in the direct pathway is equivalent to boosted expected benefit for that particular action, increasing the probability for that action to be selected in the future, i.e., reinforcement learning. Likewise, facilitating action plan transmission in the indirect pathway through paired stimulations would lead to aversive learning of the paired action because of the boosted expected cost.

The cost-benefit model predicts that exogenous stimulations of the two pathways during a choice period or a decision window (i.e., while multiple action plans are competing) should directly and instantly influence the competition and thus the impending choice. Exogenous activations of the direct pathway for a particular action during a choice period should boost the expected benefit of that action and thus the probability for that action to be chosen in the present trial. Activating the indirect pathway should decrease the probability of that action. In fact, transient pre-movement optogenetic activation of striatal neurons induced choice bias between two action alternatives in the predicted way (Tai et al., 2012). Unilateral pre-movement activation of the direct pathway caused bias towards the contralateral choice (e.g., left hemisphere direct pathway activation increased the frequency of rightward turning), whereas indirect pathway activation caused bias away from the contralateral choice. It has been shown that striatal neurons are normally active in movements of the body parts on the contralateral side (Nambu, 2011). Thus, in the framework of the cost-benefit model, the optogenetic experimental results could be explained as follows: unilateral activations of the direct pathway artificially boost the expected benefit for the contralateral movement, whereas unilateral indirect pathway activation boost the expected cost for the contralateral movement. Notably the authors of this optogenetic study proposed that activation of direct pathway striatal neurons mimics an increase of the action value of the contralateral choice and indirect pathway activation mimics a decrease of the action value, which is consistent with the cost-benefit model (Tai et al., 2012).

The cost-benefit model is also compatible with motivational effects of dopamine related drugs. Dopamine exerts opposite effects on striatal neurons in the two pathways through two different dopamine receptors: predominantly D1 receptors in the direct pathway versus predominantly D2 receptors in the indirect pathway (Gerfen et al., 1990). Dopamine binding to D1 receptors enhances dendritic excitability and facilitates glutamatergic signal transmission in direct pathway striatal neurons, whereas dopamine binding to D2 receptors inhibits glutamatergic signal transmission in indirect pathway neurons (Cepeda et al., 1993). Therefore, the cost-benefit model predicts that the overall effect of boosted tonic dopamine, up to a certain level, is to amplify the expected benefit while attenuating the expected cost. Confirming the prediction, rodents under higher tonic levels of dopamine selected energetically costly actions more frequently (Niv, 2007; Floresco et al., 2008). Selective effects of dopamine on the indirect pathway are also consistent with the cost-benefit model. Humans with more D2 receptors tended to choose energetically costly actions more frequently than those with fewer D2 receptors, most likely because dopamine can more effectively suppress indirect pathway activation in the presence of more D2 receptors, discounting cost effects (Treadway et al., 2012). Through the same mechanism but in the opposite direction, rodents injected with a D2-antagonist avoided energetically costly movements more often than before the injection (Salamone et al., 2007).

## Future directions

While the cost-benefit model presented here can account for a wide spectrum of findings, it is nevertheless a working hypothesis to be further tested and elaborated by future experiments. First, double dissociations between the direct and indirect pathways in terms of the benefit versus cost evaluation have yet to be shown. For instance, the model can be tested by directly correlating neural activity in the direct and indirect pathways with the expected benefit versus cost. Contrary to the classical model, the cost-benefit model predicts that vigorous movements would elevate indirect pathway activity because of their associated high cost. Concurrently direct pathway activity would be elevated to the extent to which there is an increased benefit associated with vigorous movements. Second, intervention of two-pathway activity during movement would affect online feedback control behaviors. For instance, amplifying direct pathway activity using D1 agonists would amplify prospective benefits, and the animal might produce more costly in-flight adjustments such as faster corrections. Third, neuromodulator signal pathways that encode cost factors and affect synaptic plasticity in the BG need to be uncovered. Phasic dopamine signals play a critical role in reinforcement learning (Schultz et al., 1997; Shen et al., 2008). However, dopaminergic neurons show heterogeneous, unreliable response to stimuli predicting punishment or effortful actions and to negative outcomes (Bayer and Glimcher, 2005; Matsumoto and Hikosaka, 2009; Gan et al., 2010). Thus, reinforcement learning likely depends on coordinated interactions among heterogeneous dopamine pathways and possibly involves non-dopamine pathways as well (Burke et al., 2012; Lammel et al., 2012). Fourth, the cost-benefit model is likely an oversimplification. A more complete picture of the cost-benefit analysis would emerge with a comprehensive understanding of the interconnections between the two pathways, the hyper-direct pathway from the cortex to the subthalamic nucleus, and structures outside the BG implicated in reward- or cost-dependent action selection such as the anterior cingulate cortex, orbitofrontal cortex, and insula (Rudebeck et al., 2006; Croxson et al., 2009). Finally, the concept of cost-benefit analysis in the sensorimotor BG may be extended to other BG functions, such as limbic and associative functions, given that other functional divisions of the BG follow similar organizational principles (Haber and Knutson, 2010). That is, evaluating positive versus negative outcomes of multiple combinations of cortical inputs via the direct versus indirect pathway to select the cortical process with the best expected outcome may be the fundamental function of the BG.

## Conflict of interest statement

The authors declare that the research was conducted in the absence of any commercial or financial relationships that could be construed as a potential conflict of interest.
